# Mistreatment of women during childbirth and its influencing factors in public maternity hospitals in Tehran, Iran: a multi-stakeholder qualitative study

**DOI:** 10.1186/s12978-023-01620-0

**Published:** 2023-05-24

**Authors:** Marjan Mirzania, Elham Shakibazadeh, Meghan A. Bohren, Sedigheh Hantoushzadeh, Farah Babaey, Abdoljavad Khajavi, Abbas Rahimi Foroushani

**Affiliations:** 1 Department of Health Education and Promotion, School of Public Health, Tehran University of Medical Sciences, Tehran, Iran; 2 Gender and Women’s Health Unit, Centre for Health Equity, Melbourne School of Population and Global Health, University of Melbourne, Carlton, VIC Australia; 3 Department of Obstetrics and Gynecology, School of Medicine, Vali-E-Asr Reproductive Health Research Center, Family Health Research Institute, Tehran University of Medical Sciences, Tehran, Iran; 4 Head of Department of Midwifery, Ministry of Health and Medical Education, Tehran, Iran; 5 Department of Social Medicine, School of Medicine, Gonabad University of Medical Sciences, Gonabad, Iran; 6 Department of Epidemiology and Biostatistics, School of Public Health, Tehran University of Medical Sciences, Tehran, Iran

**Keywords:** Maternal health care, Mistreatment, Respectful maternity care, Quality of care, Maternity ward, Labour and childbirth

## Abstract

**Background:**

Mistreatment during labour and childbirth is a common experience for many women around the world. This study aimed to explore the manifestations of mistreatment and its influencing factors in public maternity hospitals in Tehran.

**Methods:**

A formative qualitative study was conducted using a phenomenological approach in five public hospitals between October 2021 and May 2022. Sixty in-depth face-to-face interviews were conducted with a purposive sample of women, maternity healthcare providers, and managers. Data were analyzed with content analysis using MAXQDA 18.

**Results:**

Mistreatment of women during labour and childbirth was manifested in four form: (1) physical abuse (fundal pressure); (2) verbal abuse (judgmental comments, harsh and rude language, and threats of poor outcomes); (3) failure to meet professional standards of care (painful vaginal exams, neglect and abandonment, and refusal to provide pain relief); and (4) poor rapport between women and providers (lack of supportive care and denial of mobility). Four themes were also identified as influencing factors: (1) individual-level factors (e.g., providers’ perception about women’s limited knowledge on childbirth process), (2) healthcare provider-level factors (e.g., provider stress and stressful working conditions); (3) hospital-level factors (e.g., staff shortages); and (4) national health system-level factors (e.g., lack of access to pain management during labour and childbirth).

**Conclusions:**

Our study showed that women experienced various forms of mistreatment during labour and childbirth. There were also multiple level drivers for mistreatment at individual, healthcare provider, hospital and health system levels. Addressing these factors requires urgent multifaceted interventions.

**Supplementary Information:**

The online version contains supplementary material available at 10.1186/s12978-023-01620-0.

## Plain language summary

Mistreatment during labour and childbirth is a common experience for many women around the world. A picture of the nature and types of mistreatment; and especially its influencing factors has not yet been identified in Iran. A qualitative approach to explore manifestations of mistreatment during labour and childbirth while learning about the factors that influence them was used for this study. It obtained this information thanks to semi-structured interviews with women, maternity healthcare providers, and managers between October 2021 and May 2022. Our findings showed that women experienced various forms of mistreatment during labour and childbirth. At individual level, e.g., providers’ perception about women’s limited knowledge on childbirth process was an influencing factor for mistreatment. At healthcare provider level, a highlighted factor was provider stress and stressful working conditions. At hospital level, e.g., staff shortages played a main role; and at national health system level, participants believed that lack of access to pain management during labour and childbirth was an influencing factor for mistreatment. These findings can provide a good platform for designing and implementing intervention programs to reduce disrespectful maternity care. It can also be used as a guide for managers and policymakers to improve the quality of services provided to women.

**Supplementary Information:**

The online version contains supplementary material available at 10.1186/s12978-023-01620-0.

## Background

According to the World Health Organization (WHO), approximately 810 women died each day from pregnancy and childbirth-related causes in 2017, and most of these deaths were preventable [1]. Policymakers and health program planners have identified the main strategy for reducing maternal mortality and morbidity as increasing coverage of health care and subsequently improving the quality of care [2]. All women deserve high-quality care during pregnancy and childbirth as a right. Equality and dignity of women and newborns should also be met [3]. The WHO (2015) published the quality of care framework for maternal and newborn health, highlighting the importance of both the provision of care and experiences of care. The WHO standards of care that were directly related to experience of care were effective communication, respect and dignity, and emotional support [4]. The experience of positive maternity care is a necessity, not a luxury. The maternity care that women's experience can influence their decisions on mode of birth, health and well-being of mother and baby and their relationship, and future health care utilization [5, 6].

Despite significant advancements in maternal and newborn health care worldwide, access to quality care is not guaranteed for many women, especially in low- and middle-income countries (LMICs). Even with availability, care may be compromised by the negative experience of childbirth, including mistreatment [7, 8]. Mistreatment of women during childbirth includes physical, sexual, verbal abuse, stigma and discrimination, failure to meet professional standards of care, poor rapport between women and providers, and health systems conditions and constraints [9]. Mistreatment is important not only in terms of the violation of women's rights but also as a public health and social justice issue [10].

Respectful maternity care (RMC)—“the care provided to all women in a manner that maintains their dignity, privacy and confidentiality, ensures freedom from harm and mistreatment, and enables informed choice and continuous support during labour and childbirth”- has been recognized as a key approach to eliminating mistreatment [11]. However, RMC is a neglected issue, as increasing evidence suggests that mistreatment has become a common experience for many women around the world; and its prevalence varies from 43% in Latin America and the Caribbean [12] to 100% in Asia (India and Iran) [13, 14].

Drivers for mistreatment during childbirth have been identified in some studies, mostly conducted in African countries. The drivers included women's lack of cooperation [15] and discrimination based on age, education, socioeconomic status [16]; type of healthcare provider [17] and their beliefs and attitudes [18]; poor rewards and motivation [19]; lack of equipment [20]; poor supervision [21]; normalization of mistreatment and violence [22]; and high costs of maternity care [8]. However, much has not yet been learned about the drivers of mistreatment in maternity care, in the light of different context-oriented cultural, social, and economic conditions, as well as from the perspectives of multiple stakeholders [23].

There are limited studies on mistreatment during childbirth in Iran; and most of them have focused on the prevalence [14, 24], development and psychometrics of instruments [25, 26] and description of midwives' and women experiences [27, 28]. To our knowledge, there was no comprehensive study that could shed light on the nature and types of mistreatment and especially its influencing factors in Iran; and this may be stunting intervention efforts. In order to address this gap, the aim of this qualitative study was to explore the manifestations of mistreatment of women during childbirth and its influencing factors in public maternity hospitals in Tehran.

## Methods

### Study design and setting

This multi-stakeholder formative qualitative study is part of an implementation research project involving the development and implementation of a context-specific intervention to reduce mistreatment during childbirth and evaluation of implementation improvement strategies. This qualitative study was conducted using a phenomenological approach, which was underpinned by a constructivist philosophical paradigm between October 2021 and May 2022 in five public teaching hospitals in Tehran, Iran. The aim of this approach is to understand the essence of phenomenon from those who perceived it [29]. Tehran has 18 public hospitals, 50 private hospitals, and 28 other hospitals (hospitals that are a subset of a specific organization such as charity, affiliated with Armed Forces, affiliated with Social Security Organization, and affiliated with Islamic Azad University). Evidence show lower quality of services provided in public hospitals than the quality in private hospitals [30]. So, in this study we decided to assess the issue in public hospitals. A total of five public hospitals with the highest childbirth rates were selected. The characteristics and names of these hospitals have been reported in Table 1.

**Table 1:** Characteristics and names of study hospitals, 2021 data

Characteristics	Hospitals (Geographical location in Tehran/ name)				
	North/ Taleghani	South/ Mahdieh	East/ Arash	West/ Hazrat Rasoul Akram	Center/ Valiasr
Health outcomes					
Total births	641	5630	5332	347	2140
Number of vaginal births	277	2533	1894	105	454
Number of caesarean births	364	3097	3438	242	1686
Number of live births	655	5696	5430	359	2208
Stillbirths	2	111	77	6	45
Maternal deaths	4	0	0	9	15
Staffing					
Number of obstetricians	8	10	8	7	18
Number of midwives	18	34	26	11	39
Number of residents	15	36	30	21	31
Capacity					
Number of labour and delivery room	3	10	9	3	0
Number of beds in labour and delivery rooms	4	10	10	3	0
Designated waiting room for family members or companions	0	0	0	0	0

Source: Department of Midwifery, Ministry of Health and Medical Education (MOHME), Iran; 2021 [31]

### Participants and sampling

The study population consisted of three groups: (a) women in the immediate postpartum ward; (b) maternity healthcare providers (obstetricians, midwives, and residents); and (c) managers at the hospital level (maternity supervisors) and the Ministry of Health and Medical Education (MOHME) (policy-makers of reproductive health programs). The inclusion criteria were as follows: women who had vaginal birth and currently lived in Tehran; obstetricians and midwives with at least 1 year of work experience; residents who had passed at least one semester (6 months) in the maternity ward; managers with at least 5 years’ experience in their role. Exclusion criteria were women who have experienced perinatal infant death. Participants were selected using purposive sampling method with maximum variation (women based on age, education and socio-economic status variations; and healthcare providers and managers based on age and work experience variations).

After initial coordination and getting approvals from the hospitals, the first author (M.M.) invited eligible women, healthcare providers and hospital-level managers, in person; and explained the study objectives to them. She also invited managers with related experiences from MOHME to the study via email. All participants expressed their written consent to participate in the study prior to the interview. They were also aware that their participation was voluntarily and they could withdraw from the study at any time.

### Data collection

The data collection method consisted of in-depth interviews using semi-structured interview guides (Additional file 1: Interview guide for women; Additional file 2: Interview guide for healthcare providers and managers). In the first section of the interviews, topics such as experiences and perceptions of mistreatment during labour and childbirth and in the second section, factors influencing mistreatment of women during labour and childbirth were explored. Also, at the end of the interviews, demographic information of the participants were asked as follow: for women: age, education, job, nationality, family income, service provider type, gravida, and number of living children; for healthcare providers and managers: age, marital status and work experience. The interview guides were pilot tested before the study in two test interviews to ensure the appropriateness of the questions, but not analyzed. Using the pilot study, some questions of the guides were rephrased.

All interviews were conducted in Persian by M.M., a PhD candidate in Health Education and Promotion who has been trained in qualitative research. She had no prior relationship with the participants. However, before starting the interview with women, she introduced herself and announced that this study was part of a PhD dissertation, and their responses would never impact on the quality of care they received. She was able to establish an intimate relationship with women, and this provided to accurately present their views. She also invited healthcare providers and managers to speak about mistreatment they have witnessed and/or heard during their work. Before starting the interview, permission for audio recording was obtained from the participants. Interviews with the women were conducted before discharge in a private room in the postpartum wards of each hospital, where they could speak in confidence about their experiences and views. Healthcare providers and managers were interviewed at their workplace (a room where only the participant and the interviewer were present). Each interview lasted between 40 and 50 min, during which field notes were taken by the interviewer. Each participant was interviewed only once. We achieved data saturation by interviewing 24 women and 36 healthcare providers and managers; until no new data and/or themes were emerged.

### Data analysis

In this study, a qualitative content analysis was used, as described by Graneheim and Lundman [32]. Data analysis was performed simultaneously with data collection in three phases of open, axial and selective coding. First, M.M. transcribed the recorded interviews verbatim in Farsi, and returned them to the participants for verification or any comments. The transcripts were uploaded to the MAXQDA 18 for analysis [33]. Then, the second author (E.Sh., female professor in Health Education and Promotion and expert in qualitative research) checked the transcripts to ensure quality. M.M. and E.Sh. coded the transcripts independently. During open coding, semantic units and the initial codes were extracted by reading the transcripts line-by-line. Any disagreements in coding were resolved through discussion. Then the codes were classified within more comprehensive categories based on their similarities and differences. During axial coding, the relationship between categories was conceptualized. In selective coding, the core theme was identified. In order to coding the data, the typology of mistreatment during childbirth developed by Bohren et al. [9] and the Ratcliffe’s framework of the risk factors of disrespect and abuse during childbirth [34] were used. Based on the mistreatment typology, women's experiences were in the categories of physical and verbal abuse, failure to meet professional standards of care, and poor rapport between women and providers. Also, based on the Ratcliffe’s framework of the risk factors of disrespect and abuse during childbirth, the extracted components were classified into four main themes: individual-level factors, healthcare provider-level factors, hospital-level factors and national health system-level factors. We translated the selected quotations in English for providing in this paper.

Lincoln and Guba criteria (1985) were used to ensure the trustworthiness of the study [35]. The lead researcher (M.M.) had a long relationship with study settings (hospitals) which helped to gain participants' trust as well as understand the research setting and context (prolonged engagement). Coded interviews were returned to three participants for feedback. Furthermore, data collection from multiple stakeholders such as women, healthcare providers, and managers (data source triangulation) provided greater credibility to the data. To ensure dependability, the interviews were analyzed independently by two authors. Conformability was obtained by reviewing and confirming the data analysis process by a researcher familiar with qualitative studies who did not participate in the study. Variation in participant selection also contributed to the transferability of the findings. The consolidated criteria for reporting qualitative research (COREQ) checklist was used to report this manuscript [36] (Additional file 3: COREQ Checklist).

## Results

A total of 60 interviews were conducted (women: 24 interviews; healthcare providers: 29 interviews; and managers: 7 interviews). Tables 2 and 3 show the demographic characteristics of the participants. Three people refused to participate in the study including a manager who did not respond to our invitation and two healthcare providers who expressed concerns about lack of time. The results are presented in two sections: first, an overview of the experiences and manifestations of mistreatment during labour and childbirth, followed by the factors affecting the mistreatment, which is the main focus of this article.

**Table 2:** Women’s demographic characteristics

Characteristics	N (%)
Number	24 (100.0)
Age (years)	
≤ 20	2 (8.3)
21–30	16 (66.7)
31–40	6 (25.0)
Education	
Illiterate	2 (8.3)
Primary	7 (29.2)
Cycle	3 (12.5)
Diploma	6 (25.0)
University	6 (25.0)
Job	
Housewife	21 (87.5)
Employed	3 (12.5)
Nationality	
Iranian	17 (70.8)
Afghan	7 (29.2)
Family income (self-report)	
Low	8 (33.3)
Middle	14 (58.3)
High	2 (8.3)
Service provider type	
Resident	20 (83.3)
Midwife	0 (0.0)
Obstetrician	0 (0.0)
Do not Know	4 (16.7)
Gravid	
1	11 (45.8)
2	8 (33.3)
≥ 3	5 (20.8)
Number of living children (including most recent birth)	
1	12 (50.0)
2–3	10 (41.7)
≥ 4	2 (8.3)

**Table 3:** Healthcare providers’ and managers’ demographic characteristics

Characteristics	Doctors	Midwives	Managers
Number	13	16	7
Age (years)			
25–34	12	6	0
35–44	1	8	0
45–54	0	2	3
≥ 55	0	0	4
Marital status			
Single	6	6	1
Married	7	10	6
Work experience (years)			
≤ 5	12	5	0
6–10	1	3	0
11–15	0	5	1
> 15	0	3	6

### Types of mistreatment experienced during labour and childbirth

Three main themes including physical and verbal abuse, failure to meet professional standards of care, and poor rapport between women and providers were emerged from the analysis of data about mistreatment experiences (Table 4).

**Table 4:** Themes, sub-themes and codes of manifestations of mistreatment during labour and childbirth

Themes	Sub-themes	Number of codes
Physical and verbal abuse	Pressure on abdomen / Fundal pressure	6
	Judgmental comments	14
	Harsh and rude language	12
	Threats of poor outcomes	8
Failure to meet professional standards of care	Painful vaginal exams	20
	Neglect and abandonment	6
	Refusal to provide pain relief	3
Poor rapport between women and providers	Lack of supportive care	11
	Denial of mobility	18

### Physical and verbal abuse

#### Pressure on abdomen / fundal pressure

Some women complained of pressure on their abdomen by healthcare providers during childbirth, calling it an “agonizing” behavior. While the providers stated that sometimes they had to use fundal pressure to save the baby’s life.

*“During childbirth, she pressed my abdomen so hard that my abdomen turned blue. I told her ‘not to push’. She said ‘be quiet and help. My ribs hurt.”* (Woman 21, 32 years old)

*“… Sometimes we use fundal pressure because the labouring woman does not cooperate; the baby's head is in the middle of her legs, but she closes her legs.”* (Resident 1, 31 years old)

#### Judgmental comments

Judgmental comments were common form of mistreatment experienced by women. This type of mistreatment took several forms, including judgmental comments about a woman’s young age and shaming women for crying out from labour pains.

*“They said to me: You, who given birth before and have birth experience, aren’t you ashamed to shout? You have to endure the pain; you did not come here to have fun.”* (Woman 13, 24 years old)

#### Harsh and rude language

Our participants believed that harsh and rude language were common types of maternity mistreatment.

*“During childbirth, when I was screaming, they said: Shut up, shut your mouth.”* (Woman 23, 24 years old)

*“Here, the cleaners also yells at us.”* (Woman 21, 32 years old)

#### Threats of poor outcomes

Some women also described being threatened of poor outcomes by the providers. However, the healthcare providers did not consider these threats to be mistreatment; they believed that these threats came from a place of caring and help, rather than malice.

*“The doctor said: ‘Madam, you do not push, your child will be handicapped.”* (Woman 2, 27 years old)

### Failure to meet professional standards of care

#### Painful vaginal exams

Most of the women interviewed complained of frequent and painful vaginal examinations. They reported that the providers performed the examination without explanation or permission.

*“I think they examined me more than twenty times. With long nails, it really agonizes. They came quickly, put on gloves, and started the examination ...”* (Woman 9, 27 years old)

*“Yes, unfortunately, women are examined a lot. For example, by the first-year resident, then the second-year resident to check the accuracy of the first one …”* (Midwife 6, 28 years old)

#### Neglect and abandonment

The narrations of the women showed that some suffered from neglect and abandonment by the healthcare providers. The providers refused to sympathize with them during labour or left them alone after birth.

*“In the labour room, no matter how much I shouted. No one paid attention to me until the birth.... When the baby was born, I was left alone again because another woman was in pain and all the doctors and midwives went to take care of her. I was very scared because there was no one by my side to help.”* (Woman 6, 18 years old)

#### Refusal to provide pain relief

Ignoring women’s requests for pain relief during childbirth has also been reported by several women:

*“She did not use painkillers for me. She said, ‘There will be four stitches; it's not worth using painkillers. Be patient.”* (Woman 23, 24 years old)

### Poor rapport between women and providers

#### Lack of supportive care

Reports from women and some midwives indicated a lack of supportive care for women during labour and childbirth. They stated that healthcare providers do not allocate time for them to have an emotional relationship with women or to provide information throughout labour and birth.

*“I was much stressed. She was my first child and was to be born sooner. Instead of explaining or encouraging me, they said, 'Shut up, does anyone cry because of childbirth?”* (Woman 12, 19 years old)

*“We do not have an emotional relationship with the mother. She likes us to explain to her, for example, what is going to happen to her and what the delivery process is like, but unfortunately we do not spend time on it at all.”* (Midwife 3, 40 years old)

#### Denial of mobility during labour

Denial of mobility during labour was also reported by most women. They were often connected to the monitors and had no right to walk or move. However, the midwives explained that residents may have to monitor women tightly because of their legal responsibilities to the health of both mother and baby. The women could have cooperated if they knew why they were restricted under specific circumstances.

*“I was not allowed to get out of bed at all. I said let me walk, but they connected that device to me and I had to lie down, and this was kind of torture.”* (Woman 14, 38 years old)

*“… Women are not allowed to move, because of tight monitoring of the labour.”* (Midwife 8, 47 years old)

### Influencing factors of mistreatment during labour and childbirth

We identified four main themes for factors influencing mistreatment during labour and childbirth: Individual-level factors, healthcare provider-level factors, hospital level-factors and national health system-level factors (Table 5).

**Table 5:** Themes, sub-themes and codes of factors influencing mistreatment during labour and childbirth

Themes	Sub-themes	Number of codes
Individual-level factors	Perception of healthcare providers about women’s limited knowledge on labour and childbirth process	12
	Untrained companions	6
	Mismatched expectations of women for care	4
	Discrimination based on ethnicity or low socioeconomic status	12
Healthcare provider-level factors	Healthcare provider stress and stressful working conditions	13
	Healthcare providers with limited personal experience of pregnancy and childbirth	6
	Neglect of midwives’ identities by doctors	9
	Poor educational contents and curriculum	6
	Low salary and lack of incentive	12
	Personal beliefs of the healthcare providers	5
Hospital-level factors	Staff shortages	17
	Lack of supervision and control	7
	Type of hospital	5
	Inadequate physical structures	4
National health system-level factors	Lack of access to pain management during labour and childbirth	6
	Perceptions about forced vaginal birth in public hospitals	4

### Individual-level factors

#### Perception of healthcare providers about women’s limited knowledge on labour and childbirth process

Healthcare providers reported poor knowledge of pregnant women about labour and childbirth processes as major factor of mistreatment. They believed that despite being free of charge, most women did not attend childbirth preparation classes, and this lack of knowledge plays an important role in their lack of “cooperation” during childbirth and mistreatment.

*“Unfortunately, many women do not have the knowledge of birth processes. If the woman knows what a normal birth is like; how long does it take; what should she do at each stage of labour; I do not need to shout at her.”* (Resident 7, 32 years old)

#### Untrained companions

Some healthcare providers believed that the companions should have received the necessary training in order to be able to help the birthing women, while they did not have enough information and interrupt unnecessarily in the childbirth process.

*“Companions are completely unaware, their interference makes us angry, and this may lead to aggression with the mother.”* (Obstetrician 5, 33 years old)

#### Mismatched expectations of women for care

Some healthcare providers considered the high levels of women’s expectations for receiving high quality services as another factor for their mistreatment. Because this factor often caused women to be abusive to the providers and ultimately to provoke sharp reactions from the providers.

*“Some women are very expectant. They expect care like private hospitals, meaning having private doctor and midwife.”* (Resident 3, 30 years old)

#### Discrimination based on ethnicity or low socioeconomic status

Both healthcare providers and women believed that being women who were not Iranian may be at higher risk of mistreatment. In Tehran, this was particularly true for Afghan women:

*“Many of the women referred to our hospital are Afghan women, some of whom do not understand our accent at all and do not cooperate well with us. Usually this causes a sharp reaction from us.”* (Midwife 9, 50 years old)

*“When I was in the delivery room, they said, ‘Afghans again, these Afghans are everywhere we go’ ... We were offended by their words. I saw that Iranian women were treated better.”* (Woman 16, 23 years old)

Women with low economic status are more likely to experience mistreatment. Because most women who go to public hospitals are in poor financial condition, they inevitably accept any kind of care from a healthcare provider. Furthermore, the level of education of women was so important that illiteracy or low education prevented them from receiving respectful care.

*“I think a group of people come here (public hospitals) who are either illiterate or financially compelled. So they tolerate any situation and their voice is not heard.”* (Hospital level manager, 55 years old)

*“Most of my friends told me not to go to X hospital. The behavior of its staff is very bad. It looks like you are a laboratory rat. But because the cost was low, I had to come here.”* (Woman 12, 19 years old)

### Healthcare provider-level factors

#### Healthcare provider stress and stressful working conditions

High anxiety and stressful working conditions of the healthcare providers can play a significant role in their way of behaving as a health staff. Some of them complained of pressure from seniors. Seniors were always under legal pressure of providing healthy childbirth outcome and they transfer the pressure to junior healthcare providers. This was also reported by some women.

*“The professors are also pressuring us. For example, when I have to take a non-stress test (NST) for a pregnant woman who has no problem at 3 o’clock in the morning, of course I get nervous. Because I can’t find the fetus’s heart, I vent my anger on the patient. ‘Pull down your pants, lady, hurry, this happens many times and the reason is that when the senior resident or professor comes, the NST should be in the patient's file.”* (Resident 12, 27 years old)

*“I think they are treating us under pressure. Cause our health matter for them and they feel responsible.”* (Woman 17, 22 years old)

Moreover, the long hour shifts of healthcare providers, especially residents, were another factor stated by the participants that created the ground for mistreatment of women by creating physical and mental fatigue.

*“A resident who has to spend a 36-hours shift cannot be expected to be kind to patient.”* (Resident 10, 28 years old)

#### Healthcare providers with limited personal experience of pregnancy and childbirth

Participants emphasized that most providers (especially resident doctors as the primary maternity providers) are young. They are often single or have not experienced pregnancy or childbirth, and both healthcare providers themselves and women believed that this lack of understanding and empathy can be accompanied by mistreatment.

*“Most (healthcare providers) are young, maybe they do not have the experience of motherhood and childbirth, and they do not understand what the pain of childbirth is?”* (Woman 3, 38 years old)

*“I had a vaginal delivery, the way I treat a pregnant woman is far different from a single resident because I experienced the pain of childbirth, and I am more patient with the sighs and groans she makes.”* (Resident 4, 33 years old)

#### Neglect of midwives’ identities by doctors

Midwives’ report showed there is no good interaction between the obstetric residents and the midwives: *“The relationship between the resident and the midwife is not very good. How can one expect respect for the patient when they do not value us?”* (Midwife 4, 37 years old).

Furthermore, most midwives stated that obstetricians and residents should work mostly in the field of surgery and high-risk clinical activities; and midwives should be responsible for caring for women during labour and birth, particularly involving empathy and low risk timely care for women. Therefore, in order to improve the quality of obstetrics care and respecting pregnant women, it is necessary to review the job descriptions of maternity ward providers in public hospitals.

*“I have no clinical responsibility as a midwife to give birth in teaching hospitals. I only do paper works. Why shouldn’t midwife control labour process?”* (Midwife 14, 40 years old)

*“Description of midwives duties in a teaching hospital should be clearly defined. We suggested that low-risk delivery be performed by midwives.”* (MOHME level manager, 51 years old)

#### Poor educational contents and curriculum

Training gaps for healthcare providers were reported as an important factor for mistreatment by participants. They believed that they have not well trained about medical ethics or the way to communicate with women during labour and birth and that it should be included in their curriculum as a separate course.

*“We do not have enough information about dis/respectful maternity care, I have only passed a communication skills training course during my studies.”* (Resident 2, 31 years old)

*“Doctors or midwives are clinically literate, but they do not know how to treat a patient respectfully. I think it is necessary to hold regular training courses for them.”* (Woman 18, 27 years old)

#### Low salary and lack of incentive

Healthcare providers believed that low salary, along with a system of punishment, instead of encouragement and reward, affected the quality of care provided by them and the quality of relationship with patients. This was also mentioned by some women.

*“When you are not paid well and you are not satisfied financially, this can automatically affect your behavior.”* (Hospital level manager, 55 years old)

*“… Maybe they misbehave because of their limited incomes and low motivation to work.”* (Woman 24, 23 years old)

#### Personal beliefs of the healthcare providers

It seems that personal beliefs of the healthcare providers were other influencing factor for mistreatment of women. Some healthcare providers believed that they were obliged to misbehave with the women for sake of their babies’ health.

*“Yes, we should frighten the woman and tell her that if she* doesn’t *push well, her baby will die. Otherwise, she will not push at all ... But I do not consider this as mistreatment because I want to help her and her baby.”* (Midwife 1, 43 years old)

### Hospital-level factors

#### Staff shortages

Most healthcare providers complained about staff shortages. For them, performing the clinical routine tasks and paper work was a priority, and caring for women with respect was not prioritized. They also noted that the patient-to-staff ratio increases their job demands, meaning that the resident or midwife is forced to perform tasks that are not defined in their area of responsibility.

*“The patient input is very high; for example, each midwife has to cover 6 or 7 patients. So, we cannot treat all of them properly.”* (Midwife 2, 39 years old)

#### Lack of supervision and control

The women’s report showed that maternity ward managers did not monitor the performance of healthcare providers. They believed that continuous monitoring of providers' performance was required to reduce disrespectful maternity care. This issue was also emphasized by some managers.

*“There is no management and supervision. If they punish the provider who mistreated, the rest will definitely perform better.”* (Woman 19, 25 years old)

*“A person, for example, maternity supervisor, should be responsible for monitoring the behavior of staff with women and have the authority to warn if someone disrespects them.”* (MOHME level manager, 55 years old)

#### Type of hospital

Women believed that the type of hospital was important in receiving quality care. They thought that women in public teaching hospitals were more likely to experience mistreatment due to high work load of staff and lower costs.

*“The more money you pay, the better they will treat you. I think a private hospital is better.”* (Woman 9, 27 years old)

#### Inadequate physical structures

Healthcare providers stated that the lack of physical space in some maternity wards poses a challenge to the privacy of pregnant women as well as the presence of a companion. Moreover, the providers complained about the lack of adequate space for their rest during long shifts.

*“We do not have a good space here. The women were separated by the curtain, which is either torn or we have to constantly push it aside so we can see the fetal heart monitor, so privacy is not respected.”* (Resident 6, 27 years old)

*“Unfortunately, we do not have space for companions; even our residents do not have a suitable place to rest in this hospital.”* (Obstetrician 13, 43 years old)

### National health system-level factors

#### Lack of access to pain management during labour and childbirth

Midwives and residents reported that pregnant women did not have sufficient options for pain management during labour, including use of epidurals.

*“In our hospital, almost no painless normal delivery is performed. It is very difficult for us to coordinate a painless normal delivery. Sometimes an anesthetist is so late that the woman gave birth.”* (Resident 9, 32 years old)

#### Perceptions about forced vaginal birth in public hospitals

Some women also complained about forced vaginal birth in public hospitals. They believed that public hospitals limited women’s abilities to express preferences for caesarean birth, and that by giving birth in a public hospital, doctors would force them to have a vaginal birth.

*“The doctor told me that this is a public hospital, we do not perform Cesarean sections. Even if you are in pain for five days, you have to endure to give birth vaginally ... one of them tore my amniotic sac, forcing me to give birth normally.”* (Woman 7, 28 years old)

## Discussion

This was a formative qualitative study using a phenomenological approach that investigated the experiences on and influencing factors of mistreatment during labour and childbirth in public teaching hospitals in Tehran from perspective of the multiple stakeholders. Our findings showed that there were multiple level factors for mistreatment in the hospitals. These findings can provide a good platform for designing and implementing intervention programs to reduce disrespectful maternity care. It can also be used as a guide for managers and policymakers to improve the quality of services provided to women.

The results of our study showed that women experienced various forms of mistreatment during labour and childbirth. While this type of care was unacceptable to all women, some healthcare providers did not consider it as mistreatment. Verbal abuse was reported by women that were consistent with finding of other studies [37, 38]. A study conducted by Shirzad et al., (2019) in Tehran to investigate women’s perspectives on health facility and system levels factors influencing mode of delivery [39]. Some women also reported physical abuse such as fundal pressure. Our findings are consistent with previous studies conducted in other settings globally, in which women experienced verbal abuse, neglect and abandonment, lack of supportive care [38, 40, 41], frequent vaginal examinations [40, 42], and denial of pain relief [40].

In Iran, the MOHME has been promoting childbirth preparation classes in public hospitals (2008) and health centers (2014) with the aim of empowering pregnant women and their families. These eight-session childbirth preparation classes are held for free of charge, in which the presence of a chosen companion (especially family members and/or spouse to prepare them for labour companionship) in two sessions is allowed [43]. However, in our study, healthcare providers' statements indicated that women's “lack of cooperation” during childbirth due to poor attendance at childbirth preparation classes led to limited knowledge about labour and childbirth process; and it led to mistreatment. First, all women have the right to respectful care regardless of their age, social, economic, ethnic, racial or other factors. Women are often called “uncooperative” when in reality their own needs, bodies and preferences are not taken care of within the health system or by healthcare providers; and it's more about lack of effective communication than that women acting poorly. Because at the same time providers are saying women are uncooperative, but acknowledge that they aren’t educating women about what to expect. Second, failure to hold the childbirth preparation classes routinely and poor supervising of their implementation could have provided the ground for poor attendance of women in classes, as well as the presence of untrained companions. The importance of attending routine antenatal care (ANC) and accessing information and preparing for childbirth to prevent mistreatment is highlighted in other studies too [8, 20, 44, 45].

The healthcare providers believed that lack of information and knowledge about the birth process of women and their companions possibly led to mistreatment. It seems that a more critical perspective to this concept would be helpful. In a qualitative evidence synthesis conducted by Shakibazadeh et al., (2018) to develop a global conceptualization of RMC, they reported that women living in high income countries (HICs) tended to receive information to help them make right decisions and participate actively in their childbirth [46]. It seems that the level of knowledge is fundamental, not to collaborate, but with the power of choice of families, especially women in childbirth; however, women in LMICs are less likely to expect personal choice and decision making over their childbirth experience [46]. This highlights the differences in cultural norms around childbirth. Globally, healthcare providers have consistently identified the necessity of raising awareness about RMC [46, 47].

The findings of our study showed women who expected high quality services were more exposed to disrespect due to communication tensions with healthcare providers. Moreover, non-Iranian women—and particularly Afghan women—experienced discrimination in the maternity care settings. Low quality care for Afghan women, including limited access to ANC and mistreatment during childbirth (especially discrimination) has been confirmed in other studies in Iran [48, 49]. Also, poor socioeconomic status and low education of women were other factors that contributed to their experience of mistreatment. These findings were consistent with studies conducted in Kenya [50], Tanzania [51], Nigeria [52] and Palestine [16].

In our study, training gaps for healthcare providers was one of the factors of mistreatment that participants emphasized on. They believed that their educational content focused on issues of ethics rather than issues of respect, independence, and patient choice. The results of our previous study also showed that healthcare providers had poor knowledge and attitudes about disrespectful maternity care [53]. Therefore, it is necessary to review the curriculum of obstetrician and midwifery education as well as in-service continuing training based on the concept of dis/ respectful maternity care. Implementing interventions based on educating healthcare providers has been suggested as an important strategy to respect patients’ rights and reduce disrespectful maternity care in other studies [54, 55].

Stressful working conditions were identified as another important factor for mistreatment. For example, some providers described how a resident might be questioned by obstetricians and a senior resident or pressured to have a healthy childbirth outcome. Simultaneously, long shifts and low salary not only discouraged residents from providing respectful care, but also reinforced feelings of anger towards the patients and the health care system. In the study by Burrowes et al., stress, high workload, and low remuneration of the providers were also described as factors for mistreatment [56].

Neglect of midwives' identities by doctors was another factor identified in our study. Conflict between obstetricians and midwives might be due to unclear roles, poor management, hierarchy issues, and lack of sufficient skills and knowledge [57]. Consistent with our findings, a systematic review about the perspective of midwives in sub-Saharan Africa showed the low position of midwives in the health system hierarchy as a driver for disrespectful maternity care [59].

Lack of supervision and control in mistreatment emerged as another factor in our findings. Women and managers believed that the performance of healthcare providers was not monitored and that deterrent mechanisms should be considered to reduce maternity mistreatment. Similarly, Taghizadeh et al. in Iran reported that there was no legal protection for women being mistreatment in maternity wards, and this influenced their decision for future births, and they preferred never to have another birth [27]. The study by Dwekat et al. showed that the lack of accountability mechanisms and monitoring system deprived Palestinian women of their right to respectful care [16].

Participants in our study also identified mistreatment as a shortage of staff, because it limited the provision of quality care due to increased job demands. Moreover, the possibility of respecting women's privacy and sometimes labour companionship was not realized due to lack of physical space in some maternity hospitals. Also, none of the hospitals we studied had a waiting room for family members or companions. These findings are similar to the previous studies that have shown that staff shortages and poor infrastructure contributed to unintentional mistreatment [16, 44, 50]. On the other hand, women in this study reported that they were more likely to experience mistreatment in public hospitals. This is in line with the results of other studies that have shown that women viewed public facilities as a place to provide low-quality care [60] and believed that they should go to private hospitals to receive quality and respectful care [39].

Since 2014, Iran MOHME has launched the policy of “promoting vaginal childbirth” as one of the nine programs of the Health Transformation Plan with the aim of promoting maternal and infant health. One of the important strategies to achieve this goal was to provide methods to relieve labour pain in public hospitals. However, the statements of the providers in our study showed that painless childbirth is not performed in any of the studied maternity wards. Due to the lack of necessary infrastructure such as equipment and space, human resources, cooperation of anesthetists and obstetricians, and training of the women and her choice, painless childbirth is not operational in Iranian hospitals and women are deprived of this right [61, 62].

Our study findings can provide a launching point for identifying and designing interventions to reduce disrespectful maternity care in Iran. These interventions should emphasize training of pregnant women and their companions by strengthening childbirth preparation classes (awareness of labour and childbirth process and coping strategies with labour pain), training of providers, encouraging and motivating and managing providers' work shifts, strengthening the position of midwives in public hospitals. Moreover, continuous monitoring of providers' performance, increasing staff numbers and improving physical infrastructures, as well as the implementation of related guidelines, including painless childbirth, should also be considered.

To date, interventions to reduce mistreatment and/or promote RMC have been designed and implemented in different settings across the globe [10, 54, 55, 63]. However, there is poor understanding of the implementation aspects of these interventions. So, future research studies should focus on providing strong evidence on potential barriers and facilitators to implementing these interventions and explore appropriate strategies to improve their implementation; in a way that helps the planners and implementers of dis/respectful maternity care interventions to achieve the greatest effectiveness and sustainability with the least challenges.

### Strengths and limitations

One of the important strengths of this study was the use of multiple stakeholders' perspectives that provided a deeper understanding of the factors affecting maternity mistreatment in hospitals in Tehran, Iran. Second, interviewing women immediately after delivery reduced the likelihood of recall bias. However, interviews in hospitals might have hindered women from freely expressing their experiences and views. To reduce this limitation, interviews were conducted in a private room (in the postpartum ward). Furthermore, all women were reassured that this study would have no effect on the care they receive. However, there were several limitations. In this study, the obstetricians are young; so, the findings on their viewpoints should be interpreted cautiously. Also, our study was conducted in public teaching hospitals in Tehran and the results may not be generalizable to all hospitals in Iran. Further studies are suggested throughout the country, as well as in non-public hospitals. Additionally, data analysis was performed by two researchers. It is recommended to use investigator triangulation, which increases the likelihood of identifying patterns and consistencies in the data and helps reduce researcher bias.

## Conclusions

Our study showed that women experienced various forms of mistreatment including physical and verbal abuse, failure to meet professional standards of care, and poor rapport between women and providers. Our study also showed that there were multiple level drivers for mistreatment at individual, healthcare provider, hospital and health system levels. Addressing these factors requires urgent multifaceted interventions.

## Supplementary Information

**Additional file 1.** Interview Guide for Women.

**Additional file 2.** Interview Guide for Healthcare Providers and Managers.

**Additional file 3.** COREQ (COnsolidated criteria for REporting Qualitative research) Checklist.

## Data Availability

The datasets generated and analyzed during the current study are not publicly available due to privacy restrictions of the participants but are available from the corresponding author on reasonable request.

## Abbreviations

WHO: World Health Organization
LMICs: Low and middle income countries
RMC: Respectful maternity care
MOHME: Ministry of Health and Medical Education
COREQ: Consolidated criteria for reporting qualitative research
NST: Non-stress test
ANC: Antenatal care
HICs: High income countries
