# Supplementary material for: Mistreatment of women during childbirth and its influencing factors in public maternity hospitals in Tehran, Iran: a multi-stakeholder qualitative study
Source: Reprod Health. 2023 May 24;20:79. doi: 10.1186/s12978-023-01620-0 (PMC10207711; doi:10.1186/s12978-023-01620-0)
Supplement: Supplementary file 1 — Additional file 1. Interview Guide for Women. [file 12978_2023_1620_MOESM1_ESM.docx]

**Additional file 1: Interview Guide for Women**

- Could you describe your experience during labour and childbirth in the hospital? Please explain to us what happened?
- Sometimes, women are mistreated or poorly treated during labour and childbirth. This mistreatment can take several forms (including physical abuse; sexual abuse; verbal abuse; stigma and discrimination; failure to meet professional standards of care; poor rapport between women and providers; and health systems conditions and constraints). Have you ever witnessed yourself or other women being mistreated during labour and childbirth? Could you give an example?
- In your opinion, what are the factors influencing the mistreatment of women during labour and childbirth? Please explain.
- Probe women-related factors (such as age, ethnicity, and education) or healthcare providers (such as staff shortages, and low salary).
- How do these factors contribute to mistreatment of maternity care? Please explain.
- In the end, is there anything else you would like to add?
