# Supplementary material for: Mistreatment of women during childbirth and its influencing factors in public maternity hospitals in Tehran, Iran: a multi-stakeholder qualitative study
Source: Reprod Health. 2023 May 24;20:79. doi: 10.1186/s12978-023-01620-0 (PMC10207711; doi:10.1186/s12978-023-01620-0)
Supplement: Supplementary file 2 — Additional file 2. Interview Guide for Healthcare Providers and Managers. [file 12978_2023_1620_MOESM2_ESM.docx]

**Additional file 2: Interview Guide for Healthcare Providers and Managers**

- As a healthcare provider/ manager, could you comment on mistreatment of women during labour and childbirth?
- Sometimes, women are mistreated or poorly treated during labour and childbirth. This mistreatment can take several forms (including physical abuse; sexual abuse; verbal abuse; stigma and discrimination; failure to meet professional standards of care; poor rapport between women and providers; and health systems conditions and constraints). Have you ever seen or heard of these types of mistreatment happening in your work? Could you give an example?
- In your opinion, what are the factors influencing the mistreatment of women during labour and childbirth? Please explain.
- Probe women-related factors (such as age, ethnicity, and education) or healthcare providers (such as staff shortages, and low salary).
- How do these factors contribute to the mistreatment of maternity care? Please explain.
- In the end, is there anything else you would like to add?
